# Tutorial for writing systematic reviews for the Brazilian Journal of Physical Therapy (BJPT)

**DOI:** 10.1590/bjpt-rbf.2014.0077

**Published:** 2014

**Authors:** Marisa C. Mancini, Jefferson R. Cardoso, Rosana F. Sampaio, Lucíola C. M. Costa, Cristina M. N. Cabral, Leonardo O. P. Costa

**Affiliations:** 1 Programa de Pós-graduação em Ciências da Reabilitação, Departamento de Terapia Ocupacional, Escola de Educação Física, Fisioterapia e Terapia Ocupacional (EEFFTO), Universidade Federal de Minas Gerais (UFMG), Belo Horizonte, MG, Brazil; 2 Laboratório de Biomecânica e Epidemiologia Clínica, Grupo PAIFIT, Universidade Estadual de Londrina (UEL), Londrina, PR, Brazil; 3 Programa de Pós-graduação em Ciências da Reabilitação, Departamento de Fisioterapia, EEFFTO, UFMG, Belo Horizonte, MG, Brazil; 4 Programa de Mestrado e Doutorado em Fisioterapia, Universidade Cidade de São Paulo (UNICID), São Paulo, SP, Brazil; 5 Musculoskeletal Division, The George Institute for Global Health, Sydney, NSW, Australia

## Abstract

Systematic reviews aim to summarize all evidence using very rigorous methods in order to address a specific research question with less bias as possible. Systematic reviews are widely used in the field of physical therapy, however not all reviews have good quality. This tutorial aims to guide authors of the Brazilian Journal of Physical Therapy on how systematic reviews should be conducted and reported in order to be accepted for publication. It is expected that this tutorial will help authors of systematic reviews as well as journal editors and reviewers on how to conduct, report, critically appraise and interpret this type of study design.

## Introduction

A systematic review can be defined as the synthesis and analysis of information focussed on scientific contributions of published studies^1^. These reviews are not limited to summarize the literature but also aim to reach new conclusions^1^. This is not a new concept as there are reviews that date back to the start of the 20^th^ century^1^^,^ 2. In the 1960s, there were already studies that integrated research results and pointed to new scientific trends, especially in social sciences, education, and psychology. The recognition of the importance of the application of the best scientific information available in the field of health care created the need to support clinical practice on evidence and consequently a gradual increase in the demand for this type of information has occurred^2^.

Evidence refers to the body of information used to confirm or refute a scientific theory or hypothesis and it is produced by a systematic process of investigation1.

Despite the cumulative characteristic of scientific knowledge, the scientific methods to synthesize evidence were only developed in the 20^th^ century. In conjunction with these advances, scientists began to recognize that organizing and assessing this cumulative scientific knowledge went beyond a simple choice of method, exposing the need for greater methodological rigor to ensure the validity of the literature reviews just as it is required of the primary studies^3^.

There is a consensus that the synthesis of knowledge is fundamental to the advance of clinical practice, research and implementation of health policies. However, synthesizing knowledge in a clear and precise manner requires specific skills and competencies. Searching and selecting the relevant studies, assessing their risk of bias, and summarizing data are some of the challenges of conducting systematic reviews^4^.

Common steps in systematic reviews: a) a clear definition of the objectives of the review; b) development of a research protocol; c) comprehensive search strategies to find all relevant studies; d) a method to assess potential risk of bias of individual studies; and e) description of data collection and summarizing the data4.

A variety of terms have been used to describe the processes of integration of evidence: research-synthesis, systematic review, integrating review, meta-analysis, etc.^1^. The growing popularity of this type of study is illustrated by the fact that, to keep up to date, many professionals choose to read literature reviews. Among the different types of studies that synthesize scientific evidence, systematic reviews follow strict assessment criteria and provide solid conclusions that can be applied to clinical practice. Furthermore, systematic reviews can identify significant gaps in knowledge and point out the need for new studies.

Guidelines, reporting guidelines and checklists aim to regulate the process of writing systematic reviews from study selection to the final structure of reporting the results^5^. The researcher must choose from among the existing rules and regulations which one is best suited to the topic to be investigated and to the journal chosen for submission of the review.

Examples of guidelines and reporting guidelines for systematic reviews: Cochrane Handbook (http://www.cochrane.org/handbook) and PRISMA (www.prisma-statement.org/), among others.

The first clinical trials in physical therapy were studies that assessed the effects of ultraviolet radiation in school children and children with respiratory problems and were published by Colebrook^6^ in 1929 and Doull et al.^7^ in 1931, respectively. The first systematic review in the field^8^ presented the effects of treatments on ankle ligament injuries and was published by Kolind-Sorensen^9^ in 1975. Since then, there has been an exponential growth of this type of study in the field of physical therapy. Thus, it has become imperative that scientific journals strive to set clear rules to assist researchers in reaching the quality required for the publication of systematic reviews and to make this scientific information available for the advancement of knowledge. To reflect this international movement and to provide stronger epistemic vigilance in research in physical therapy and other health-related areas, the Brazilian Journal of Physical Therapy (BJPT) has prepared the following tutorial. It aims to qualitatively align its systematics reviews, with attention to the conditions and limits of the concepts and techniques employed in this process.

## What is systematic review and meta-analysis?

According to the Cochrane Collaboration Handbook^10^, a systematic review is a secondary study that aims to gather similar published or unpublished studies and critically assess their internal validity and gather them in a statistical analysis whenever possible. The systematic review also aims to minimize bias using explicit and precise methods. The statistical method used to integrate the results of the studies included in the systematic reviews is called meta-analysis^8^.

The terms meta-analysis and systematic review are often used incorrectly or interchangeably. Data aggregation in a meta-analysis does not mean that the individual studies have been carefully analyzed. Systematic reviews can be undertaken with or without meta-analysis. The difference between systematic review and meta-analysis is very important because it is always possible to systematically review a group of data (with criteria to assess the bias risk of the studies included in the review), however it can sometimes be inappropriate or even misleading to conduct data aggregation of the results of independent studies.

In general, the synthesis that results from a systematic review provides better evidence on the subject in question, such as the effects of an intervention on a specific outcome, the incidence of a disease or the accuracy of a diagnostic tests, among other topics.

In a systematic review, it is necessary to: formulate a clear review question; determine the sources and methods of study selection, such as databases and search strategies; select studies with similar methods; assess potential sources of bias and describe the methods of testing the validity of the selected studies; prepare syntheses for presentations/publications (both qualitative, i.e. descriptions of the studies, and quantitative, i.e. meta-analyses, when appropriate)10.

Systematic reviews are considered secondary studies because they summarize the information from multiple publications, such as treatment and prevention studies (randomized controlled trials - RCTs), prognostic (cohort) studies, diagnostic accuracy studies, etiologic studies, among many others. The most common are systematic reviews of the effects of an intervention. The quality of this type of review must be ensured so that health professionals, patients and regulating bodies can make more assertive decisions.

This tutorial will emphasize the treatment/prevention reviews that use RCTs. In this type of study, the participants have been randomized to one of two (or more) treatment groups. An example of this type of review is the assessment of the effectiveness of an exercise program (i.e., strengthening exercises) and other treatments (i.e., thermotherapy, electrical stimulation, taping etc.) for patients submitted to arthroscopic partial meniscectomy. The search was conducted for studies published between 1950 and 2013, and 18 RCTs were included in the review, but only six were selected for statistical analysis, i.e. meta-analysis. The authors concluded that the abovementioned outpatient physical therapy procedures combined with instructions for exercise at home improved knee function, according to patient reports, and knee flexion and extension range of motion compared to outpatient physical therapy alone^11^.

There are well-defined guidelines for reporting RCTs (www.consort-statement.org) and systematic reviews (www.prisma-statement.org/ and Cochrane Collaboration Handbook10).

In sum, every systematic review involves a careful analysis of the risk of bias of the studies and some systematic reviews employ meta-analysis. The analysis of risk of bias considers both internal and external validity and the statistical analyses used in each of the included studies. Meta-analysis, in turn, is a systematic and rigorous statistical procedure that can be reproduced by other researchers and allows the combination of the results of different studies. The meta-analysis adjusts and weighs the results according to the sample size of each primary study. It can also be adjusted for other factors, such as the risk of bias of each study.

## Types of systematic review

It is important to point out that many clinical questions can be synthetized in one systematic review. The most common and popular systematic review aims to measure the effect of an intervention (i.e., systematic review of RCTs). However, systematic reviews can be very useful for summarizing other review questions such as: prevalence^12^, incidence^13^, prognostic factors^14^, diagnostic accuracy^15^, cost-effectiveness^16^, risk factors^17^, definition of research terms^18^, cross-cultural adaptations of questionnaires^19^, measurement properties of instruments^20^, and systematic reviews of qualitative studies^21^^,^ 22.

As a result of this range of possibilities, the author must identify the appropriate study design for each type of systematic review. Although it might seem obvious, this has been identified as one of the main problems in the process of peer review of systematic reviews submitted to the BJPT. Therefore, the author must always choose the ideal design for each type of scientific question.

The most adequate design choices would be RCTs for reviews aimed at measuring the effects of intervention, prospective (longitudinal) cohort studies for prognostic reviews or risk factor reviews or cross-sectional studies for prevalence reviews.

In some types of questions, different study designs may be adequate. For example, a diagnostic accuracy systematic review allows the inclusion of case control studies, cross-sectional studies and, in some cases, even clinical trials. However, the combination of different designs is more of an exception than a rule.

## Essential items in a systematic review

Systematic reviews differ greatly with regards to the review question and the eligibility criteria. Certain rules apply to some reviews but not to others. Nevertheless, there are a few items that are essential and should be present in all reviews. They are as follows:

1. Clear definition of the review question: a good review is not the one that answers a lot of questions, but the one that answers specific questions clearly and with the least possible bias. Thus, the definition of the review question is essential. The PICO (Patient, Intervention, Comparison and Outcomes) process is a good approach to framing a question for intervention systematic reviews. For example: "*are manual therapy techniques associated with an exercise program (Intervention) better than exercise alone (Comparison) for reducing pain and disability (Outcomes) in adult patients with low back pain (Patients)?*" The research question can be framed using the PICO structure in a flexible manner, e.g. mentioning the comparison later in the review: "*what are the effects of joint mobilization (Intervention) in improving range of motion, pain, and disability (Outcomes) in patients treated with ankle immobilization (Patients)?*" In this case, the comparison groups are any type of control group. Another flexible use of the PICO structure is when the review does not assess the effect of the intervention; in this case, the "I" is attributed to the focus of the study (see types of systematic review above).

For a clear framing of the review question:

1. It is vital that the research clearly define the **intervention (or study focus)**, the **outcomes** and the **population** of interest. These three items are of fundamental importance in framing a review question
2. It is recommended that, when formulating the question in an intervention study, the author specifically identify the intervention (i.e., resistance exercise, instructions to carers, etc.), instead of naming the intervention(s) tested in the study as the profession or area (e.g., physical therapy, rehabilitation)
3. Other types of systematic review other than intervention studies must follow the same steps for framing a review question: clear, direct, and well formulated questions
4. A well-formulated question will also guide several aspects of writing a systematic review, including search strategies, study eligibility, data extraction and conclusions

2. Determining eligibility criteria: after formulating the review question, the author must determine a priori the inclusion and exclusion criteria for the studies that will be considered eligible for the review. This includes study design, intrinsic characteristics of each study (i.e., sample, types of treatment, duration of symptoms, equipment used, etc.), and dates and language of publication. Ideally, studies should not be excluded based on time of publication, risk of bias or language.

3. Ensuring all eligible studies were retrieved^8^^,^ 23: this is one of the hardest tasks in a systematic review, because it should aim to include ALL available evidence. Therefore, searches must be performed in multiple databases. That often constitutes a problem for some researchers, as access to these databases is not always free. A few examples of paid databases are EMBASE, CINAHL, MEDLINE, and PSYCHINFO. It is worth noting that only 14 physical therapy journals are indexed in PubMed (the free version of MEDLINE) and, therefore, there is a high probability that studies relevant to physical therapy will not appear in searches performed only in free-access databases.

The same reasoning can be applied to the language of the databases: many authors search in local databases, such as SCIELO and LILACS. Nevertheless, these databases only index studies published in Portuguese and/or Spanish, which account for less than 2% of the world's scientific literature^24^.

In addition to the careful selection of databases, another essential item in finding all studies is to formulate an efficient search strategy. A search strategy requires adequate descriptors that change according to each database and its Boolean operators (AND, OR, NOT). An efficient strategy captures all potentially eligible studies (i.e., search with high sensitivity), but also eliminates irrelevant studies (i.e., search with high specificity).

4. Clear presentation of aspects related to data extraction: after determining the eligible studies, it is essential that the author clearly present the data that will be extracted from each study as these data will determine the results of the review.

5. Assessment of risk of bias of eligible studies: there are several scales that assess the risk of bias of various types of study design. This bias is related to the systematic error that can occur. Examples include: selection, performance, detection, attrition, reporting, among other sources of bias. It is essential that the conclusions of a systematic review be weighted according to the risk of bias presented in the studies.

The instruments available for assessing the risk of bias in the RCTs included in a systematic review include the PEDro scale^24^ and the Cochrane risk of bias tool^25^. The PEDro scale rates the methodological quality and statistical reporting of RCTs. It consists of the following items: Inclusion criteria and source of participants; random allocation; concealed allocation, baseline comparability regarding the most important prognostic indicators; subject blinding, therapist blinding, assessor blinding; >85% follow up for at least one key outcome; intention-to-treat analysis; between-group statistical comparison for at least one key outcome; and point estimates and variability measures for at least one key outcome^24^. The Cochrane risk of bias tool considers random sequence generation; allocation concealment; blinding of participants and personnel; incomplete outcome data; selective reporting; group similarity at baseline; co-interventions; similarity of interventions; intention-to-treat analysis; time of outcome assessment, and other sources of bias^25^^,^ 26. Clearly, both instruments are very similar, with the exception of the risk of performance and detection bias are only presented in the Cochrane risk of bias tool.

6. Synthesis of results: there are two ways of presenting the results of a systematic review: 1) meta-analysis (this topic is described in the next section) or 2) descriptive. The presentation of the results must take into account not only the results of the studies but also the risk of bias of each of the included studies.

7. Discussion: A systematic review should include a discussion on the following items^27^:

1) statement of principal findings, 2) strengths and weaknesses of the study, 3) strengths and weaknesses in relation to other studies, discussing important differences in results, 4) unanswered questions and future research, and 5) meaning of the study: possible explanations and implications for clinicians and policymakers.

## Meta-analysis

The purpose of the meta-analysis is to obtain the combined effect of a treatment^28^. When conducting a meta-analysis, it is important to observe the homogeneity of the procedures adopted by the authors of the RCTs, that is, the characteristics of the studies, such as: characteristics of the intervention(s) to be assessed (e.g. similarities between intensity, frequency, and duration), and how the variables or clinical outcomes were measured or classified. If the review is conducted correctly, with a search strategy that is consistent with the review question and that generates a reasonably complete group of valid studies on the topic and without bias, the meta-analysis will also deal with the intended question. In contrast, if the search strategy is conducted incorrectly or if the studies present biased results, the problems of the review will not be corrected with a meta-analysis^29^.

When reading a meta-analysis, it is important to understand its four parts^28^^,^ 29 shown below.

### The presentation of the results of a meta-analysis must allow the reader to **understand** 29 **:**

1. What type of measure was used?

2. What does the forest plot show? 

3. What does the combined effect (pooled effect) indicate? 

4. Is it valid to combine the studies?

1. Different types of measure in a meta-analysis

Because the meta-analysis calculates the statistical synthesis of the effect of interest, it is important to understand the nature of the data that are combined, whether categorical or continuous. In addition, the effect in each study can be presented in different ways (i.e., using mean differences, weighted mean differences, odds ratio, relative risk, among other effect estimates).

The outcome variables of individual (primary) studies can be continuous (i.e., range of motion in degrees, maximal inspiratory pressures in mmHg) or categorical (i.e., classification of the severity of a disease, presence or absence of improvement in disability, number of patients who improved, etc.).

2. The forest plot^10^

The graphic representation of the measures of the effect of each individual study, as well as the combined effects, is called forest plot. The term "forest" was created because the graph looks like a forest of lines. The central vertical line of the forest plot indicates when there is no statistically significant difference between the groups. The points represent the mean differences of each study and the horizontal lines, the confidence intervals surrounding the mean differences. The diamond represents the **combined (pooled)** effect of all of the study effects of a specific comparison analyzed by the meta-analysis. The interpretation of a forest plot is very simple: if the diamond or the confidence intervals touch the central line, there is no between-group significant difference. However, if the diamond does not touch the central line, there is a statistically significant difference between the analyzed groups. Every forest plot also contains the estimates, which allows the readers to determine whether the observed differences are clinically significant. Finally, the forest plot can show, at the author's or journal's discretion, the weight of each study in the final combined effect, as well as present statistical data on the heterogeneity of the data.

**Figure 1:**
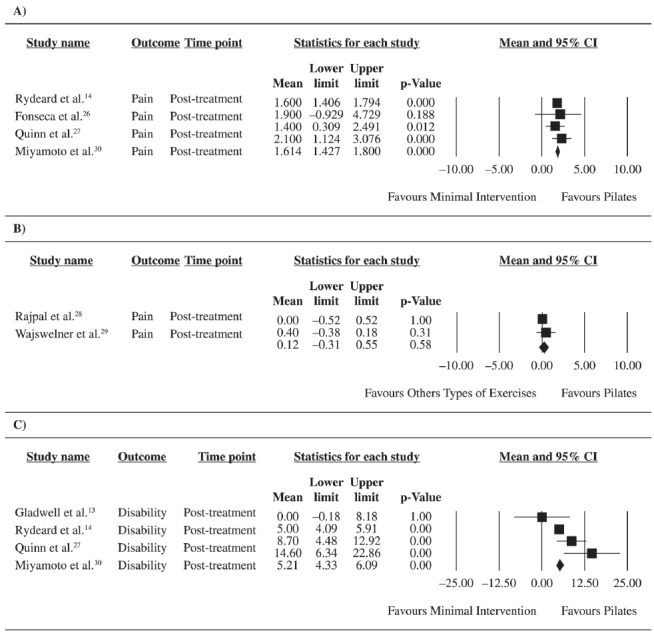
Forest Plots published in Miyamoto et al.^30^, pag. 525. Reprinted with permission.

Three forest plots recently published in the BJPT^30^ are shown in Figure 1. These forest plots are part of a systematic review that compared the effects of Pilates exercises in patients with low back pain. Graphs A and C compare Pilates with minimal intervention (i.e., educational booklets) for the outcomes pain intensity and disability, respectively, and graph B compares Pilates with other types of exercise. The diamond does not touch the central line in graphs A and C, but it does in graph B. The conclusion of these meta-analyses is that Pilates is more effective than minimal intervention, but it is not more effective than other types of exercise in patients with low back pain.

3. Pooled effect

The pooled effect represents the combined effect of all individual studies in each comparison. This effect takes into consideration the effects of each study, and the estimated confidence interval is weighted by their sample size.

4. Is it valid to combine the studies?

It is not always possible to combine the results reported by the studies^10^^,^ 28. The review should only combine studies that are homogeneous from a clinical point of view (i.e., similar interventions with similar dosages), that used similar outcome measures, that used similar control groups, and had homogeneous data. If any of these premises is violated, a meta-analysis must not be conducted.

There is a debate on the assessment of heterogeneity of the studies in a meta-analysis. We recommend reading the book by Borenstein et al.28 and the Cochrane Handbook10. Cochrane has a free software program (http://tech.cochrane.org/revman/download) for systematic reviews and meta-analyses.

## Assessing the quality of a systematic review of the literature

The assessment of the quality of a systematic review includes various parameters, such as the quality of the selected studies (i.e., risk of bias) and its methodological homogeneity or heterogeneity (i.e., similarity of the sample characteristics, outcome tools and measurements, forms of administration of intervention, statistical heterogeneity, etc.), as well as structural characteristics (i.e., clarity and pertinence of the review question, adequacy of search strategy, clarity and validity of conclusions, etc). Authors interested in conducting a systematic review should seek information in order to conduct the review with the highest methodological standard and in a way that meets the quality criteria.

Accordingly, the BJPT has strived to maintain the quality of the systematic reviews it publishes. Between 2012 and 2014, 77 systematic reviews were submitted and only seven were published. Three are currently under review, 27 were filed because they did not meet the journal's standards, and 40 were rejected. That means that 87% of submitted reviews did not meet the quality criteria for publication in the BJPT in this time period. According to analysis, the main reasons for rejection by the editors were: methodological problems in the process and description of the review (including non-compliance with the PRISMA checklist^31^); the study is entitled systematic review, but does not have the structural requirements of a systematic review; the study does not make a contribution to the area or is outside the scope of the BJPT. Similarly, *Physical Therapy Journal* performs an initial assessment of all systematic reviews that it receives and the criteria for immediate rejection without being assigned to full peer review are: 1) the review is not based on the clear and objective question; 2) it is not clinically useful or it is outside the scope of physical therapy; 3) the searches for eligible studies are not considered broad enough to convince the editors that all potentially eligible studies were included; 4) the risk of bias of the eligible studies was not measured or the risk of bias was not taken into consideration in the data interpretation; 5) it has serious methodological flaws; 6) a similar review has been published recently and a new one is not justified; and 7) presentation of a meta-analysis in the absence of a systematic review.

For some time, the rules of the BJPT have suggested that authors follow PRISMA^31^ guidelines, which contain recommended items for the reporting of systematic reviews. These recommendations describe, in detail, 27 items that must be presented in systematic reviews, in addition to a checklist to be submitted with the manuscript.

The PRISMA checklist31 (Portuguese version)32 can be accessed on: http://www.scielo.br/img/revistas/rbfis/2012nahead/pt_038anx01.jpg

In a study that aimed to analyze the reporting of systematic reviews published in the area of physical therapy in Portuguese, Padula et al.^32^ found that there was little influence of the PRISMA recommendations in most of the reviews, even after its publication in 2009^31^. The authors point out that this is not related to the methodological quality of these systematic reviews, given that the PRISMA guidelines pertain to the reporting of reviews and not to the assessment of their methodological quality. The fact that most of the published systematic reviews do not meet PRISMA standards is a warning to the scientific community about the transparency of the methods and results of these reviews and, consequently, about the extent to which these results should influence clinical practice. Since most of the recommendations are not followed, it is likely that many reviews are published based upon the results of the review, which indicates publication bias^32^.

The assessment of the quality of systematic reviews of health interventions can be performed with the AMSTAR (Assessment of Multiple Systematic Reviews) tool^33^^,^ 34. AMSTAR is a valid instrument^35^ that consists of 11 items that assess the process of study search and selection, characteristics and scientific quality of included studies, appropriate use of methods to combine findings of studies, assessment of the likelihood of publication bias, and documentation of conflict of interests. A study that assessed the quality of systematic reviews of oral health interventions in a Brazilian journal found that the methodological quality of the systematic reviews is still very low^36^. Thus, the process of analysis of systematic reviews submitted to journals should include a methodological quality assessment using the AMSTAR^36^ tool.

In sum, we recommend that the authors use guides such as the Cochrane Handbook, the AMSTAR tool, and the PRISMA checklist when preparing research projects for systematic reviews, as well as during research and reporting. Attention to these guidelines will increase the quality of the reviews and consequently lead to peer review in the BJPT. Ultimately, the guidelines can result in more precise and balanced conclusions that will help in the decision-making process of physical therapists and other health professionals.

## Concluding remarks

The process of systematic reviews aims to gather, examine, and systematically assess the results of studies that aim to answer a well-framed clinical question. The final manuscript can be a systematic review of the literature, with or without meta-analysis, and the quality will depend on the procedures involved in its preparation and on the transparency in its reporting. As happens with other publications, systematic review reporting varies, challenging readers to evaluate the strengths and weaknesses of the conclusions.

To collaborate with the quality of the systematic reviews of the BJPT, the present tutorial provided an overview of this type of study and endeavored to highlight the fact that methods and guidelines are evolving and becoming increasingly specialized, therefore their importance should not be underestimated. Strict methods of systematic reviews improve the quality, scope, and applicability of results and contribute to better care and the development of guidelines for clinical practice, and the advancement of research and health policies.

Following this publication, the BJPT will include PRISMA in the process of submission of systematic reviews. This change expresses not only the journal's attention to the transparency and consistency of the findings reported in this type of study but also reinforces its constant initiatives to enable its authors, editors, and reviewers. It is expected that these tools will strengthen the process of peer review and improve the evidence made available by the studies published in the BJPT.
